# GRK5 Deficiency Leads to Selective Basal Forebrain Cholinergic Neuronal Vulnerability

**DOI:** 10.1038/srep26116

**Published:** 2016-05-19

**Authors:** Minchao He, Prabhakar Singh, Shaowu Cheng, Qiang Zhang, Wei Peng, XueFeng Ding, Longxuan Li, Jun Liu, Richard T. Premont, Dave Morgan, Jeffery M. Burns, Russell H. Swerdlow, William Z. Suo

**Affiliations:** 1 Laboratory for Alzheimer’s Disease and Aging Research, Kansas City Veterans Affairs Medical Center, Kansas City, MO 64128, USA; 2 Department of Cognitive Sciences, Beijing Institute of Basic Medical Sciences, Beijing, 100850, P.R. China; 3 Department of Neurology, Sun Yat-sen Memorial Hospital, Sun Yat-sen University, Guangzhou, 510120, P. R. China; 4 Department of Medicine, Duke Univ. Med. Center, Durham, NC 27710, USA; 5 The Johnnie B. Byrd Alzheimer’s Center & Research Institute, Tampa, FL 33620, USA; 6 Deptment of Molecular Pharmacology & Physiology, University of South Florida, Tampa, FL 33620, USA; 7 Department of Neurology, University of Kansas Medical College, Kansas City, KS 66170, USA; 8 Department of Molecular and Integrative Physiology, University of Kansas Medical College, Kansas City, KS 66170, USA; 9 The University of Kansas Alzheimer’s Disease Center, Kansas City, KS 66160, USA

## Abstract

Why certain diseases primarily affect one specific neuronal subtype rather than another is a puzzle whose solution underlies the development of specific therapies. Selective basal forebrain cholinergic (BFC) neurodegeneration participates in cognitive impairment in Alzheimer’s disease (AD), yet the underlying mechanism remains elusive. Here, we report the first recapitulation of the selective BFC neuronal loss that is typical of human AD in a mouse model termed GAP. We created GAP mice by crossing Tg2576 mice that over-express the Swedish mutant human β-amyloid precursor protein gene with G protein-coupled receptor kinase-5 (GRK5) knockout mice. This doubly defective mouse displayed significant BFC neuronal loss at 18 months of age, which was not observed in either of the singly defective parent strains or in the wild type. Along with other supporting evidence, we propose that GRK5 deficiency selectively renders BFC neurons more vulnerable to degeneration.

Cognitive impairment in Alzheimer’s disease (AD) and in other related disorders is a major health challenge for society because of its high prevalence and disproportionally high costs of care and because of a lack of disease-modifying treatments^1^. The tremendous efforts of AD researchers have significantly improved understanding of this disease; however, the particular subset of neurons responsible for the progressive memory loss in AD remains unclear.

Among many of the hypotheses concerning the pathogenesis of AD, the cholinergic hypothesis is the only hypothesis that links memory loss to a particular subset of neurons, although it faces challenges as any other hypotheses. The cholinergic hypothesis synthesized biochemical, electrophysiological and pharmacological evidence accumulated over a decade and postulated a relationship between significant cholinergic dysfunction and memory loss in the aged and demented central nervous system^2^.

One of the challenges to the cholinergic hypothesis is why basal forebrain cholinergic (BFC) neurons are more vulnerable than other neurons to degeneration. Multiple explanations have been proposed, including differential levels of intra-BFC (as opposed to non-cholinergic) neurofibrillary tangles, β-amyloid (Aβ) protofibrils, neuronal nitric oxide synthase, calcium dysfunction, and nerve growth factor signaling defects^3^,^4^,^5^,^6^,^7^. Aside from the intriguing evidence supporting each explanation, these studies all share some important limitations. For example none of these studies have satisfactorily demonstrated whether these differential degenerative changes in the BFC neurons are the cause or the consequence of BFC neurodegeneration, nor have any of their findings been recapitulated in an animal model *in vivo*.

In this regard, our efforts were not initially focused on explaining BFC neuronal vulnerability. In trying to understand why Aβ-pretreated cells become hyperactive, we discovered the dysfunction of G protein-coupled receptor kinases (GRKs) in AD, including GRK5 deficiency^8^. Subsequent explorations in GRK5-knockout (GRK5KO) models have revealed that GRK5 deficiency accelerates AD pathogenesis^9^,^10^,^11^. However, the relevant mechanistic studies have failed to identify any effect of GRK5 deficiency on the functions of several suspected G protein-coupled receptors (GPCRs) *in vivo*^12^, except for muscarinic cholinergic receptors, which were initially reported in 1999^13^. For the latter, we have further narrowed the affected receptors to the inhibitory G_i_-coupled M2/M4 receptors^14^. Considering the unique link between AD, GRK5 deficiency, and cholinergic dysfunction, we performed an unbiased stereological quantification of BFC neurons in GRK5-deficient Swedish β-amyloid precursor protein (APP) transgenic (Tg2576) mice, which we named GAP mice^10^,^11^,^12^. Our results were highly consistent and suggested that GRK5 deficiency leads to selective BFC neuronal vulnerability.

## Results

### BFC neuronal loss in aged GAP mice

**Figure 1:**
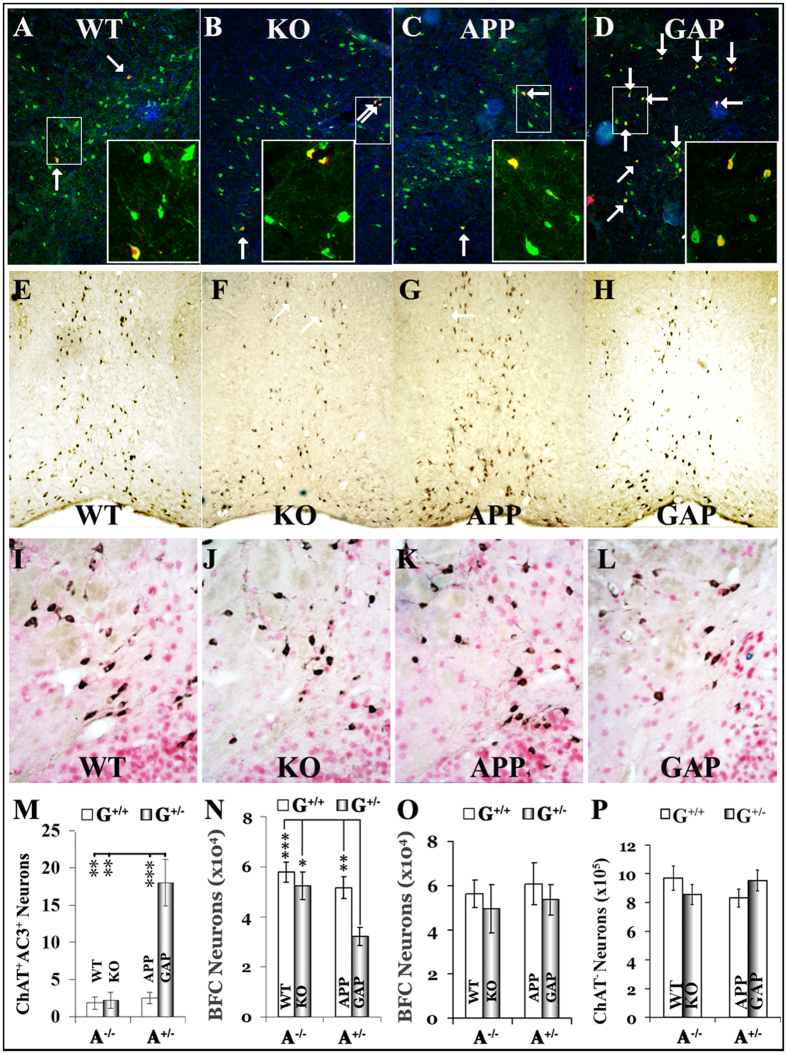
BFC neuronal loss in aged GAP mice. GAP mice were euthanized at either 12 or 18 months of age as indicated, and the brains were collected and embedded into multi-brain blocks via services from NeuroScience Associates as previously described^9^. The cryosections were stained with the indicated antibodies. ChAT = green (**A**–**D**) or brown (**E**–**H**); AC3 = red; DAPI = blue. Panels (**A**–**D**) show examples of AC3^+^/ChAT^+^ neurons (arrows) in the vertical limb of the diagonal band of Broca (VDB) in 18-month old of WT (*n* = 5), KO (*n* = 4), APP (*n* = 6), and GAP (*n* = 6) mice, respectively. A small frame in each panel (**A**–**D**) is enlarged in the corresponding insert. The numbers of apoptotic BFC neurons ((**M**), quantified as previously described^54^) were significantly increased in the GAP mice but not in the KO or APP mice. **p < 0.01 and ***p < 0.001 compared with the indicated groups. Panels (**E**–**H**) show the representative images of ChAT^+^ neurons at the low magnification (10x) in the medial septum (MS)/VDB regions of 18-month old of WT (*n* = 6), KO (*n* = 6), APP (*n* = 6), and GAP (*n* = 6) mice, respectively. Panel (**N**) shows the stereological data of total ChAT^+^ neurons in the entire basal forebrain, which reveals a significant reduction in the total number of BFC neurons in the GAP mice (55.5% of WT, ***p = 0.0007 compared with WT, **p = 0.006 compared with APP, and *p = 0.011 compared with KO) but not in the KO or APP mice. Panels (**I**–**L**) show examples of NeuN (red)/ChAT (brown) double-staining in the NBM of 18-month old of WT (*n* = 5), KO (*n* = 4), APP (*n* = 5), and GAP (*n* = 4) mice, respectively. Stereological quantification of the ChAT^−^/NeuN^+^ non-cholinergic neurons (panel (**P**)) revealed no differences between these mice in the NBM, nonCPu or CPU sub-regions. Panel (**O**) shows the stereological counting of total ChAT^+^ neurons in the entire basal forebrain of 12-month old of WT (*n* = 6), KO (*n* = 7), APP (*n* = 7), and GAP (*n* = 7) mice, which revealed no significant difference between any groups.

To quantitatively investigate BFC neuronal loss, we employed two parallel approaches. First, we used double immunofluorescent (IF) staining against choline acetyltransferase (ChAT) and active caspase-3 (AC3) to systematically determine whether there were more AC3^+^/ChAT^+^ double-positive neurons in the basal forebrains of GAP mice. We found that all four strains, wild type (WT, G^+/+^A^−/−^), GRK5KO (KO, G^+/−^A^−/−^), APP (G^+/+^A^+/−^), and GAP (G^+/−^A^+/−^), displayed AC3^+^/ChAT^+^ double-positive neurons, though the numbers in the 18-month-old female GAP mice were 7–10 times higher than those in the female littermates of WT, KO, or APP mice (Fig. 1A–D,M). To confirm this finding, we employed an unbiased stereology technique and quantified the total number of cholinergic (ChAT^+^/NeuN^+^) and non-cholinergic (ChAT^−^/NeuN^+^) neurons in the basal forebrains of these mice. We found that, compared with the WT mice, GAP mice lost 44.5% of their BFC neurons, whereas the number of cholinergic neurons in the WT, KO and APP mice was not significantly different (Fig. 1E–H,N). Moreover, the numbers of non-cholinergic (NeuN^+^/ChAT^−^) neurons in the basal forebrains were not significantly different for any of the four strains (Fig. 1I–L,P). The altered number of cholinergic, but not non-cholinergic, neurons in the GAP mice strongly suggests that this neuronal loss is selectively limited to cholinergic neurons. Notably, the significant reduction in BFC neurons in the GAP mice was observed in only the 18-month-old female GAP mice. When the same experiments were performed in 12-month-old female mice, no significant BFC neuronal loss was observed in the GAP mice or in any of the other strains (Fig. 1O). Together, these quantitative analyses revealed significant BFC neuronal loss in the aged female GAP mice but not in the age-matched female GRK5KO or APP mice.

### Exaggerated BFC axonopathy in GAP mice

**Figure 2:**
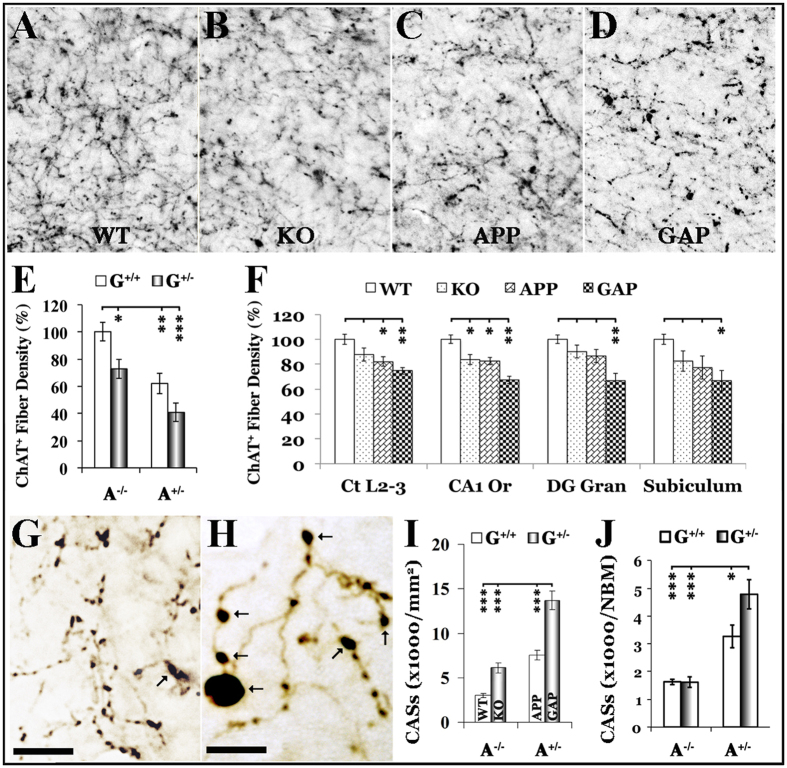
Changes in CFD and CASs in GAP mice. CFD and CASs in GAP mice were quantified either systematically or stereologically as specified. Panels (**A**–**D**) show representative ChAT^+^ fibers in the stratum oriens of the hippocampal area CA3 in 18-month-old WT, KO, APP, and GAP mice, respectively. Panel (**E**) shows the systematic quantification results of CFD in the stratum oriens of the hippocampal area CA3 in 18-month-old mice. Panel (**F**) show the systematic quantification results of CFD in the sub-regions as indicated from 12-month-old mice. Ct L2-3 = frontal cortex layer II-III; CA1 Or = hippocampal CA1 stratum oriens; DG Gran = dentate gyrus granular layer. Panels (**G**,**H**) provide examples of normal cholinergic axonal varicosities or dilations (diameter <3 μm) and abnormal cholinergic axonal swellings or CASs (diameter >3 μm, pointed arrows) in WT and GAP mice, respectively. The scale bars in panels (**G**,**H**) are 10 μm. Panel (**I**) shows systematic quantitative results of CASs in the CA3 region of an 18-month-old sample. Panel (**J**) shows stereological quantification (rare event protocol) results of CASs in the NBM of 4-month-old mice. *p < 0.05, **p < 0.01, and ***p < 0.001 for the indicated comparisons.

In addition to the BFC neuronal loss, we also quantified changes in cholinergic axonal swellings (CASs) and cholinergic fiber density (CFD) in these animals. In the 18-month-old mice with significant BFC neuronal loss, the GRK5KO, APP and GAP groups all displayed significantly decreased CFD and increased CASs, although GAP mice showed the most profound changes (Fig. 2A–E,G–I). Furthermore, although we did not observe significant BFC neuronal loss in the 12-month-old females, CFD decreased significantly in most subregions of the hippocampus and in layers 2–3 of the frontal cortex in GAP mice (Fig. 2F). To a lesser degree, 12-month-old APP mice also displayed a significant decrease in CFD in the stratum oriens of the hippocampal CA1 region and in layers 2–3 of the frontal cortex, whereas 12-month-old GRK5KO mice showed a significant decrease in CFD in only the stratum oriens of the hippocampal CA1 region. We have yet to observe any significant CFD decrease in animals younger than 12 months old. However, a significant increase in CASs in the nucleus basalis of Meynert (NBM) was revealed in 4-month-old APP and GAP mice, though 4-month-old GRK5KO showed no change in CASs at all (Fig. 2J). Moreover, the increase of CASs in the GAP mice was significantly higher than that in the APP mice. Therefore, consistently with the BFC neuronal loss, the GAP mice displayed significantly more severe and earlier cholinergic axonopathy than the parent strains. Statistically significant (p < 0.01) interactions were also found for both CASs and CFD, which suggest that GRK5 deficiency synergistically interacts with Swedish mutant APP in the GAP mice and promotes BFC neurodegeneration.

### Earlier cognitive impairment in GAP mice

**Figure 3:**
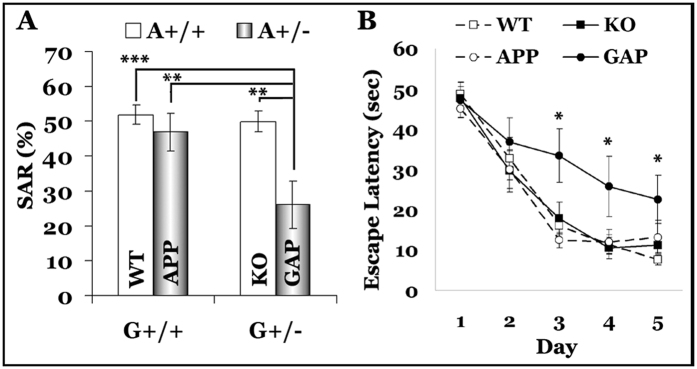
Behavioral deficits in GAP mice. Six-month-old female WT (*n* = 14), KO, (*n* = 16), APP (*n* = 14), and GAP (*n* = 10, this group began with 12 mice, with two mice excluded in the swimming screening task because they kept circling in the water) mice were behaviorally assessed with a battery of tasks as detailed in the methods. No behavioral deficits were found in the KO and APP mice, but the GAP mice were found to be cognitively impaired at this age (7 months old when the cognitive tasks were performed), as revealed by the Y maze alternation (**A**) and MWM (**B**) tasks. **p < 0.01 and ***p < 0.001, as indicated in panel (**A**) and *p < 0.05 compared with WT mice in panel (**B**).

At the behavioral level, both the GAP and APP mice were cognitively impaired at 12 and 18 months of age, with no detectable difference between the strains, whereas the heterozygous GRK5KO mice were not cognitively impaired at either age. At 7 months old, however, the GAP mice exhibited cognitive impairment in the Y-maze alternation test and the Morris water maze (MWM) task, whereas neither of the parent strains showed cognitive impairment at this age (Fig. 3). In addition, the radial arm water maze task also showed a trend of cognitive decline for GAP mice, though not statistically significant because the WT group had a rather large standard deviation (not shown). It is worth noting that the swimming screening task has excluded two GAP mice from the behavioral tasks because these two mice kept circling rather than swimming in the water. Aside from this, no sensorimotor dysfunction was found for any of these mice. Taken together, the behavioral assessments revealed that the GAP mice are cognitively impaired earlier than the APP mice, which is in agreement with the pathological observations. Nevertheless, significant cholinergic axonopathy with CASs can occur long before the detection of cognitive impairment.

Notably, GRK5 deficiency alone is sufficient to cause mild cognitive impairment in homozygous mice at 18 months^9^ but not in the heterozygous mice at the same age^12^. However, even if the strength of the GRK5 deficiency is not sufficient to cause cognitive impairment, it is sufficient to make the animals more vulnerable to cognitive impairment from additional insults, such as the over-expression of Swedish APP in the GAP mice. To this end, our studies also revealed that GRK5 deficiency is sufficient to render the animals more susceptible to intermittent hypoxia-induced cognitive impairment^15^. Therefore, these pathological and behavioral data together suggest that GRK5 deficiency facilitates both cognitive and BFC neuronal vulnerability.

### Suppressed neuronal defenses and cAMP signaling in GRK5-deficient HT22 cells

**Figure 4:**
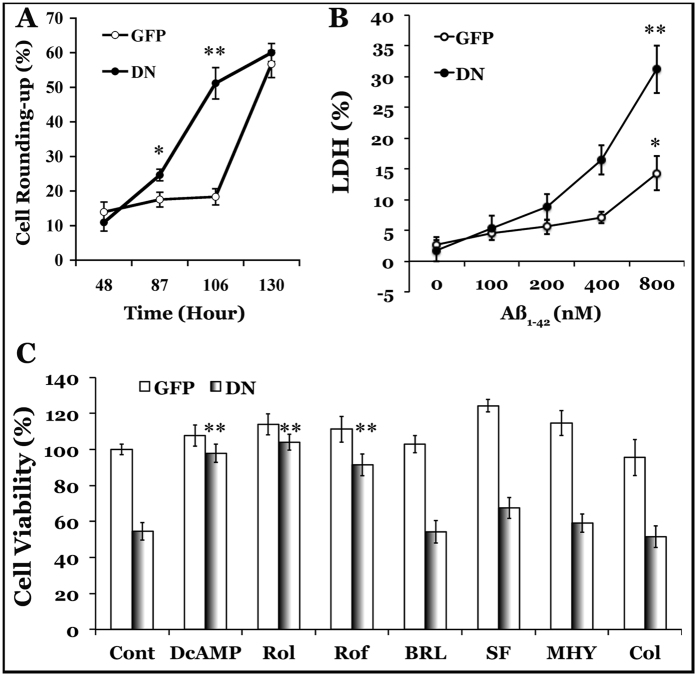
GRK5 deficiency leads to HT22 cholinergic neuronal vulnerability. HT22 cells that stably expressed dnGRK5GFP/M2 (DN) and GFP/M2 (GFP) were placed in differentiation medium for 48 h. (**A**) Cells were cultured without any treatment or medium refreshment to assess spontaneous degeneration. The cells were periodically photographed (15 random fields/well or a minimum of 300 cells/well) to calculate the rate of cell rounding. *p < 0.05 and **p < 0.001 compared with GFP. (**B**) Cells were exposed to increased concentrations (100–800 nM) of freshly solubilized recombinant human Aβ_1–42_ for 24 h to assess neurotoxicity by LDH release. The cytotoxicity is expressed as the percentage of LDH release versus the sum of LDH from the medium and the cell lysate of GFP control cells. *p < 0.05 and **p < 0.01 for dose-dependent effects in the indicated cells, as revealed by one-way analysis of variance (ANOVA). A significant (p < 0.05) interaction was revealed between DN and Aβ by multivariate analysis of variance (MANOVA). (**C**) Cells were cultured with the indicated drugs for an additional 48 h to determine whether any of the treatments prevented the spontaneous degeneration of GRK5-deficient HT22 neurons. Cell viability was measured using an MTS assay. The treatments were DcAMP (dibutyryl-cAMP, 250 μM), Rol (rolipram, 5 μM), Rof (roflumilast, 5 μM), BRL (BRL-50481, 20 μM), SF (SF1670, 10 μM), MHY (MHY1485, 5 μM), and Col (colivelin, 1 pM). **p < 0.01 for the interactions between DN and the indicated treatment, as analyzed by separate two-way ANOVAs.

To understand how GRK5 deficiency leads to cholinergic neuronal vulnerability, we employed our previously established *in vitro* GRK5-deficient model. HT22 cells are immortalized murine hippocampal neuronal cells^16^, that express ChAT, release acetylcholine (ACh) and possess cholinergic neuronal properties^14^,^17^ after differentiation. To establish a GRK5-deficient cholinergic neuronal model, we stably co-expressed dominant-negative GRK5 and humanized green fluorescent protein (GFP) fusion construct (dnGRK5GFP) and human muscarinic receptor-2 (M2) in HT22 cells to establish the dnGRK5GFP/M2/HT22 and control GFP/M2/HT22 cell lines^14^. Observing the GRK5-deficient HT22 cholinergic neurons revealed that they displayed increased vulnerability to spontaneous degeneration (Fig. 4A) and to Aβ toxicity (Fig. 4B). Moreover, the increased vulnerability of GRK5-deficient HT22 neurons was significantly corrected by directly treating the cells with a cell-permeable analog of cAMP (dibutyryl-cAMP) or with selective phosphodiesterase 4 (PDE4) inhibitors (rolipram and roflumilast). In contrast, no significant interactions were detected between GRK5 deficiency and treatment with the selective PDE7 inhibitor, BRL-50481, the PTEN (phosphatase and tensin homolog) inhibitor SF1670, the mTOR (mammalian target of rapamycin) activator MHY1485, or the neuroprotective peptide and STAT3 (signal transducer and activator of transcription 3) activator colivelin, even though SF1670 and MHY1485 slightly increased the cell viability for both the GRK5-deficient and control HT22 cells (Fig. 4C). Therefore, these results strongly indicate that suppressed cAMP signaling may mediate the increased vulnerability associated with GRK5 deficiency.

**Figure 5:**
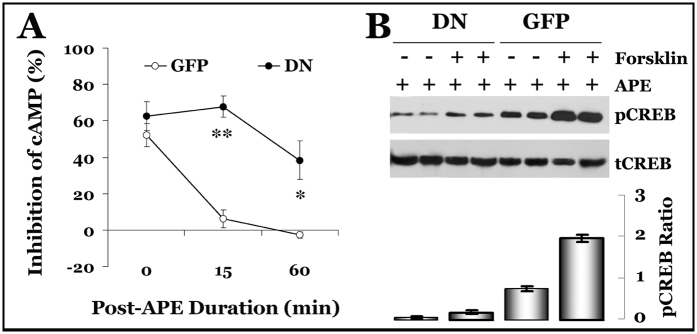
Prolonged inhibition of forskolin-induced cAMP in dnGRK5GFP/M2/HT22 cells. (**A**) The dnGRK5GFP/M2/HT22 (DN) and GFP/M2/HT22 (GFP) cells were pretreated with the M2 agonist APE (250 μM) for the duration indicated prior to being exposed to 10 μM forskolin for an additional 10 min. cAMP levels were measured using the CatchPoint fluorescent cAMP assay kit. The reduction in cAMP, calculated as a percentage of the level in non-APE-treated controls, is plotted as shown. *p < 0.01 and **p < 0.001 compared with the GFP control. (**B**) Cell lysates were analyzed in parallel for pCREB and tCREB levels by Western blotting, and the pCREB ratios were also plotted (*n* = 3) below each group as indicated.

To confirm that the cAMP signaling pathway is indeed suppressed, we directly measured changes of cAMP levels in the dnGRK5GFP/M2/HT22 cells. We found that forskolin-induced cAMP production in the GFP/M2 control HT22 cells was inhibited for only a few minutes after stimulation with the M2 agonist arecaidine propargyl ester (APE); however, this inhibition of cAMP production persisted for a much longer period of time (hours) in the GRK5-deficient HT22 cells (Fig. 5A). Consistently with the cAMP decrease, we also observed a decrease in the levels of phosphorylated cAMP response element-binding protein (pCREB) vs total CREB (tCREB) in the GRK5-deficient HT22 neurons (Fig. 5B). These results are in complete agreement with the neurotoxicity data and suggest that the persistent suppression of cAMP signaling in the GRK5-deficient HT22 cholinergic neurons leads to disrupted cellular defenses and increased neuronal vulnerability.

## Discussion

Memory deficits affected 46.8 million people worldwide in 2015. BFC projection to the limbic system is fundamental to memory function^18^,^19^. Selective BFC neurodegeneration is critically involved in memory deficits. One important question is who are more likely to suffer from memory deficits? Our results in this study suggest that GRK5-deficient subjects are more susceptible because their BFC neurons are vulnerable to degeneration.

Neurons are naturally equipped with intrinsic defense mechanisms. Neurodegeneration results from an imbalance in the intrinsic defense and extrinsic insults, in the form of decreased defense, increased insult, or both. The cAMP/CREB signaling pathway has been implicated in controlling neuronal cellular defenses^20^,^21^. In this study, we found that GRK5-deficient HT22 neurons display persistently suppressed cAMP/CREB signaling, which may have disrupted their neuronal defenses, thus making the GRK5-deficient HT22 cells more susceptible to spontaneous degeneration and to Aβ-induced toxicity.

Although the only difference between the dnGRK5GFP/M2/HT22 and GFP/M2/HT22 cells is the over-expression of dnGRK5, both cell lines also artificially over-express M2 receptors. Moreover, as detailed in the methods, all the cell culture experiments were performed in medium containing the muscarinic agonist APE or oxotremorine-M (Oxo-M) for activating M2 receptor. These conditions ensure that the inhibition of cAMP production originates from the G_i_-coupled inhibition of adenylyl cyclases (ACs) via M2 activation, which has only a brief effect in the control cells, compared with a persistent effect in the GRK5-deficient cells. It is the persistent inhibition of cAMP signaling that really suppresses neuronal cellular defense, whereas this persistency is a result from the impaired M2 receptor desensitization.

Once activated, most, if not all, GPCRs undergo deactivation or desensitization, which is the primary function of GRKs^22^,^23^. In fact, different GRK members may share substrates, which permits mutual compensation when a particular GRK member is deficient. However, this compensation is limited as revealed *in vivo* in the GRK knockout mice. For GRK5, the impairment of M2/M4 receptor desensitization is the only functional loss discovered^13^,^14^ since the generation of these knockout mice in 1999^13^. Therefore, the GRK5 deficiency appears to have a very narrow but highly selective impact *in vivo*, namely, on the cholinergic system. More specifically, because M2/M4 receptors are primarily presynaptic autoreceptors in memory circuits^24^,^25^, their impaired desensitization leads to prolonged or persistent signaling. This extended signaling first causes inhibition of ACh release, as we have previously demonstrated^14^, whereas the long-lasting effect appears to be the persistent inhibition of ACs^26^,^27^, which in turn inhibits cAMP signaling and decreases cholinergic neuronal defenses^20^,^21^. The latter effect explains why the BFC neurons in GRK5-deficient mice are particularly more vulnerable and why they are susceptible to cognitive impairments triggered by the over-expression of Swedish APP in GAP mice or by intermittent hypoxia^15^, as mentioned earlier.

Beyond the phenomena and the mechanisms revealed, this study provides the first transgenic animal model that does not only recapitulate the BFC neuronal loss that is typical of human AD^2^, but also perfectly complement the cholinergic hypothesis. Although there are many transgenic animal models for AD, a common problem is that most of them do not show significant neuronal loss, except a few that utilized aggressive transgenic tools by either over-expressing multiple familial AD (FAD) mutations or by crossing with transgenic lines carrying genetic defects with no clear relation to AD. For example, 5xFAD mouse (Tg6799) takes five FAD mutations and APP751SL/PS1KI mouse requires four FAD mutations together to drive a phenotype of neuronal loss^28^,^29^. Yet, it remains to be established whether these models displays BFC neuronal loss. In comparison, by incorporation of GRK5 deficiency that is known to be an AD-related factor^8^,^12^,^30^, it recapitulates the typical AD change of BFC neuronal loss, thus validating the cholinergic hypothesis, and enabling the relevant mechanistic and therapeutic studies.

It is worth mentioning that the incidence of AD is higher in women than that in men, and this gender characteristic has been replicated in the transgenic models as well for both the APP and GRK5KO mice^31^,^32^. For this very reason, the gender variation was taken out in the experimental design by using only the females as mentioned in the methods. Nevertheless, we did compare the levels of the BFC neuronal loss between 18-month old male and female GAP mice, and found that the females showed significantly lower BFC neuronal numbers than that in the males. Therefore, the BFC neuronal loss in GAP mice is no surprise as being a gender related phenotype.

Aside from mechanisms and animal modeling, this study verifies the importance of GRK5 deficiency in AD. Although we have previously shown that Aβ accumulation can cause functional GRK5 deficiency *in vitro*^8^, no population-based studies have been performed to determine whether all or any subpopulation of AD patients exhibit GRK5 deficiency. Therefore, this study warrants such human population-based studies with the objective of determining whether GRK5 deficiency exists in cognitively impaired or vulnerable populations with not only mild cognitive impairment and AD but also others such as OSA and Parkinson’s dementia.

At theoretical level, the results of this study complement the cholinergic hypothesis and suggest including the upstream cholinergic neurodegeneration as part of the therapeutic targets for the root cause of the cholinergic dysfunction. The cholinergic hypothesis has overwhelming supportive evidence but also faces challenges. Pathological evidence from autopsy samples of very late-stage AD patients supports a significantly more profound loss of BFC neurons than other types of neurons in AD^33^,^34^. Cholinergic hypofunction or reduced cholinergic activity is also widely documented in late AD^2^,^34^, given the Nun study found no decrease in cholinergic enzyme activity in early AD^35^. Experimental ablation of BFC neurons via immunotoxin to p75^NTR^ reproduces similar memory loss in animals, which support the role of the cholinergic system in memory^36^,^37^,^38^,^39^. Pharmacological manipulations also support a critical role for the cholinergic system in memory^2^,^40^,^41^. The cholinomimetic strategy driven by the cholinergic hypothesis eventually led to 4 out of 5 FDA-approved drugs for AD^2^,^42^. However, these cholinesterase inhibitors (ChEIs) eased only some of the symptoms without modifying the disease process^43^, leading to disappointment with and questioning of the cholinergic hypothesis.

Cholinomimetic therapeutic approaches such as ChEIs can only preserve ACh released into the synaptic cleft for a longer period of time, compensating for the low cholinergic activity at the postsynaptic level. This approach was not designed to prevent the upstream root cause of cholinergic hypofunction, namely, cholinergic neurodegeneration. From this perspective, cholinomimetic drugs have been successful in easing patients’ symptoms, and there is no reason to expect them to slow down disease progression in the first place. Therefore, we argue that the failure of the cholinomimetic approach in modifying the disease is not adequate to question the validity of the cholinergic hypothesis. Moreover, based on the findings of the present study, we now know what renders BFC neurons more vulnerable, and the therapeutic strategies in coincidence with the cholinergic hypothesis are to include the upstream cholinergic neurodegeneration as part of the targets. More specifically, the use of APE4 inhibitors and/or M2 antagonists to combat the BFC vulnerability should be tested in GRK5-deficient subjects.

In addition, selective degeneration of a particular subset of neurons is a common theme for many neurodegenerative disorders, such as for substantia nigra dopaminergic neurons in Parkinson’s disease, spinal cord motor (cholinergic) neurons in amyotrophic lateral sclerosis, and of course BFC neurons in AD^44^. Selectively affecting one subpopulation of neurons must be the result of a unique pathogenic process. Such selectivity not only determines the disease phenotype but also demands specific therapeutic approaches to tackle the unique pathogenic pathway. However, the mystery behind these selective vulnerabilities has been puzzling for decades.

From this study, nevertheless, we have learned a new way that a selective neuronal vulnerability can occur, that is the disruption of intrinsic neuronal defenses by GRK5 deficiency. We noticed that there exist other similar phenomena. For example, there are highly selective impacts for GRK6 deficiency on dopamine receptors^45^, GRK2 deficiency on adrenergic receptors^46^, and GRK3 deficiency on odorant receptors^47^. It will be interesting to see whether similar mechanisms play any roles in the vulnerability of these particular subsets of neurons.

Overall, the knowledge of GRK5 deficiency leads to selective BFC neuronal vulnerability will open new horizon to further validate the cholinergic hypothesis and to determine if preventing the upstream cholinergic neurodegeneration will slow down the disease progression.

## Methods

### Animals

GRK5KO mice, generated by targeted deletion of exons 7 and 8 of the *grk5* gene as detailed previously^13^, were crossed with APP (Tg2576) mice to produce WT (GRK5^+/+^/APPsw^−/−^, abbreviated as G^+/+^/A^−/−^), GRK5KO (G^+/−^/A^−/−^), APP (G^+/+^/A^+/−^) and GAP (G^+/−^/A^+/−^) mice for this study. All mice were backcrossed to the C57/BL6 background for at least 8 generations. Genotyping was performed using tail DNA as previously described^10^. All procedures for using these animals were approved by the Kansas City Veterans Affairs Medical Center Institutional Animal Care and Use Committee, and the experiments were carried out in accordance with the approved guidelines.

### Behavioral Assessments

All behavioral tests were performed in our behavioral lab division, which is equipped with the ANY-maze Video Tracking System along with Stoelting mouse behavioral battery devices (Wood Dale, IL). After being transferred to the behavioral division, the mice were habituated to the operator and the room environments for two weeks before testing. The behavioral tasks comprise a battery of tests that provide an extensive analysis of sensorimotor function, anxiogenic tendencies, and mnemonic performance. The procedures were the same as we recently described^15^. For all behavioral testing, the operator was blind to the experimental variables of genotype and treatments. The following tasks were evaluated in the indicated order including (1) Swimming Screening test for excluding subjects inappropriate for tasks requiring swimming; (2) Open Field to evaluate spontaneous activity and exploratory behavior; (3) Balance Beam to evaluate vestibular and general motor balance; (4) String Agility to evaluate agility and grip capacity; (5) Elevated plus maze to evaluate level of anxiety; (6) Elevated platform to evaluate level of anxiogenic tendency; (7) Y Maze to evaluate spontaneous alternation behavior and spatial working memory; (8) Morris water maze evaluates reference (spatial) learning and memory; and (9) Radial arm water maze to measure spatial reference memory.

### Tissue processing

All animals were anesthetized via intraperitoneal injection with ketamine (285 mg/kg) and xylazine (10 mg/kg), perfused with cold phosphate buffered saline (PBS) followed by 4% paraformaldehyde. Their brains were removed and post-fixed overnight with 4% paraformaldehyde. The brains were infiltrated with sucrose solution until they sank completely and then surface dried and coated with mounting gel before being cryopreserved at −80 °C. Alternatively, randomly selected brains from each group were shipped to NeuroScience Associates for multi-brain embedding and sectioning. The brains were cut along the coronal plane at a thickness of 25 μm in a continuous series and collected into 24 series groups with options to select multiple intervals as necessary. The sections were preserved at −20 °C in cryopreservation solution (30% glycerol, 30% ethylene glycol in 0.1 M PBS) before staining.

### Immunohistochemistry (IHC)

The cryopreserved sections in each group were transferred to 0.1 M phosphate buffer (PB), free floating in 6-well plates, for IHC staining with a polyclonal antibody raised against human ChAT (1:1000 dilution, Millipore, CA, USA). The sections were washed in Tris-buffered saline (TBS) and then incubated in 0.3% H_2_O_2_ in methanol for 30 min, rinsed 3 times in TBS, incubated for 30 min in TBS containing 0.25% Triton X-100 and 3% bovine serum, incubated with the primary antibody for 48 h (goat-anti-human ChAT diluted in TBS containing 1% Triton X-100 and 1% bovine serum), washed 3 times, incubated with biotinylated bovine anti-goat secondary antibody (Santa Cruz Biotechnology, TX, CA, 1:500) for 1 h, washed 3 times, incubated with the avidin-biotin–peroxidase complex (ABC Elite kit standard; Vector Laboratories, CA, USA) and finally developed using an enhanced ImmpactDAB substrate solution (ImmpactDAB kit, Vector Laboratories, CA, USA) according to the manufacturer’s recommendations. For double staining with the neuronal marker NeuN, the ChAT-stained sections were further stained with a monoclonal anti-NeuN antibody (Santa Cruz Biotechnology, TX, USA), followed by the ImmPRESS alkaline phosphatase polymer-based reagent and VectorRed substrate kit according to the manufacturer’s instructions (Vector Laboratories, CA, USA). This double-staining method resulted in a two-colored profile: brown for ChAT and red for NeuN. IF staining was performed as described previously^9^. For triple staining of NeuN/ChAT/AC3, we used mouse mAb-NeuN (1:500)/donkey pAb-Mouse-IgG-Alexa350 (1:600), goat pAb-ChAT (1:300)/donkey pAb-Goat-IgG-Alexa488 (1:800), and rabbit pAb-AC3 (1:300)/donkey pAb-Rabbit-IgG-Texas Red (1:600).

### Unbiased stereology

**Figure 6:**
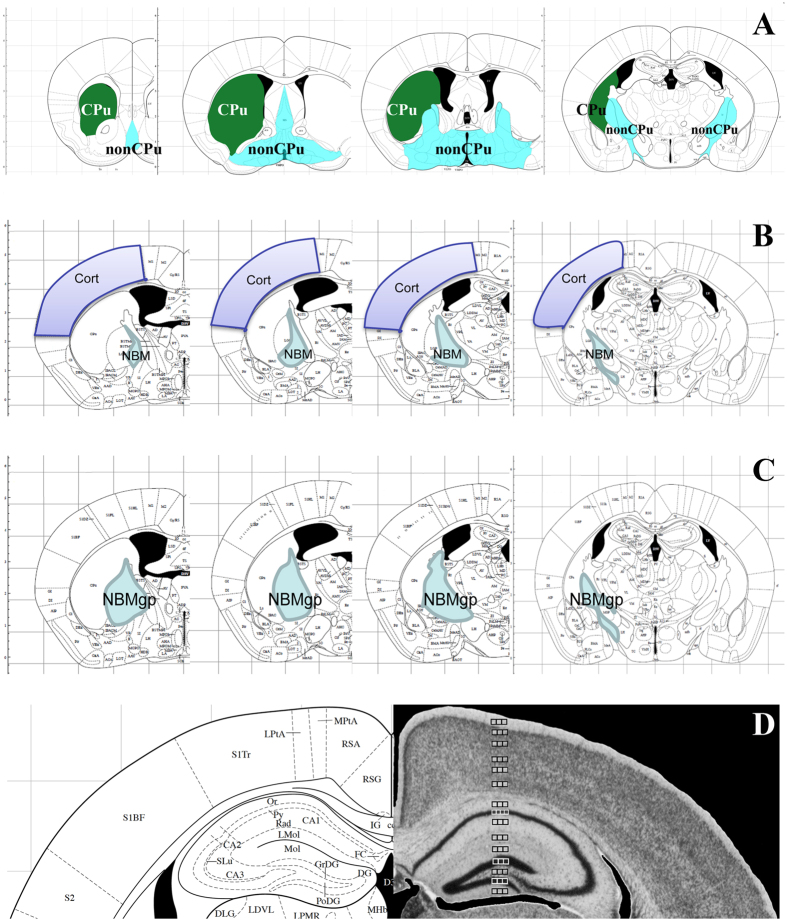
Diagrams of coronal sections delimitating the borders of regions used for stereological analysis of BFC neurodegeneration. To quantify the total numbers of cholinergic neurons (ChAT^+^/NeuN^+^) and non-cholinergic neurons (ChAT^−^/NeuN^+^), the basal forebrain was divided into two sub-regions, the CPu and the rest of the basal forebrain (nonCPu), as indicated (**A**). The neuronal counts were performed separately in the CPu and nonCPu regions to avoid potential variations due to defining boundaries of various sub-regions of the basal forebrain because the boundaries of the CPu are readily visible at low magnification (4×). The total number of neurons in the CPu and nonCPu together was used to represent the values for the entire basal forebrain. Nevertheless, to complement the potentially reduced specificity and/or sensitivity due to the inclusion of the rather large area of structures within the nonCPu, we quantified the neuronal numbers within the NBM as well (**B**). To analyze the CFD, the rostral somatosensory cortex up to bregma −1.70 mm was used to represent the cortex (**B**) in addition to the hippocampus, which is very easy to identify (not shown). The analysis of the CASs in 18-month-old mouse brains revealed very severe changes and inconsistency among various groups; therefore, we included the globus pallidus along with the NBM (labeled as NBMgp) to avoid variations due to possible differences in outlining the ROIs (**C**). Panel (**D**) depicts the sampling schemes for quantification of CFD within the cortex and hippocampus. Due to large variations among different layers of the structures, the quantification was performed in each layer of the ROIs. However, rather than sampling the entire structure, only three or four random square frames within each layer in each section were chosen to represent the average values for each of the particular structures.

We performed the stereology using a Leica AF6000 microscope (Buffalo Grove, IL) equipped with an x/y/z movement-sensitive stage and controlled by Stereologer software (Stereology Resource Center, Inc., St. Petersburg, FL). Stereologer contains most commonly used protocols, including the standard fractionator sampling, rare event, and Space Balls protocols that were used for this study. The unbiased stereological quantification is achieved by counting positive cells/structures in a known fraction of the sections that pass through a region of interest (ROI)^48^,^49^. Because the largest variation in stereology comes from defining the boundaries of an ROI that has no clear boundary (i.e., the medial septum), we divided the whole basal forebrain into two easily distinguishable areas, the caudate putamen (CPu) and rest of the nucleus basalis (nonCPu). An additional sub-region focus was the NBM. The corresponding sampling schemes are depicted in Fig. 6. To quantify the numbers of ChAT^+^ and NeuN^+^ neurons, a systematic random series of every 8^th^ (200-μm interval) section throughout the entire structure was taken for stereological counts. For each of the selected sections, the ROI was outlined at low magnification (4× objective), and the outlined region was measured at high magnification (63× dry) using a systematic random design with known dissector counting frames. The average section thickness was measured, and 2 μm guard heights at the top and bottom of each section were excluded. Optical counting rules were used to count the cells, and the examiners were blind to the identities of the samples. The counting criteria were ChAT^+^/NeuN^+^ (cholinergic) or ChAT^−^/NeuN^+^ (non-cholinergic) immunoreactivity in the cell body with a neuronal phenotype. For rare events (i.e., CASs in the NBM), every positive structure inside the frame was counted, rather than using fractionator sampling. Again, NBM was outlined at low magnification, and the CASs were identified at high magnification (63× dry) from the varicosities, based on whether their diameters were larger than 3 μm, as described previously^50^. For CFD quantification, we used either the method described by Aznavour^51^ or the Space Ball dissector method in Stereologer and analyzed cholinergic fiber length per unit of volume according to the recommendations of the software. The ROI volume was analyzed using Cavalieri’s principle. The cholinergic fiber density was calculated by dividing the total fiber length by the volume of the ROI. The sampling scheme was evaluated for the coefficients of error of the individual estimates and the cross-validation (group estimates of error), and the effects of interest (age, genotype, or treatment) were analyzed^52^.

### Cell culture and biochemical assays

The HT22 cells were a generous gift from Dr. David Schubert (The Salk Institute, La Jolla, CA)^16^. The sub-lines of dnGRK5GFP/M2/HT22 and GFP/M2/HT22 were established previously^14^. These cells were maintained in Dulbecco’s modified Eagle’s medium (DMEM) supplemented with 10% fetal bovine serum (FBS) and differentiated in NeuroBasal medium (Invitrogen, Carlsbad, CA) containing 1× N2 supplement for 24–48 h before use, as described previously^53^. For spontaneous degeneration, the differentiated HT22 neurons were maintained in the original differentiation media for the entire course of the experiment without refreshing, though APE (250 μM) or Oxo-M (50 nM) was added to all of the cells at the beginning of the experiment (48 h or as indicated after differentiation). The cells were periodically photographed (15 random non-overlapping fields/well or a minimum of 300 cells/well) to calculate the rate of cell rounding. For the MTS and LDH assays, the relevant cell viability/cytotoxicity assay kits (Promega, Madison, WI) in a 96-well plate format were used, and the manufacturer’s instructions were followed. Western blotting procedures were detailed previously^8^,^53^. For the cAMP assay, the dnGRK5GFP/M2/HT22 and GFP/M2/HT22 cells were differentiated in 96-well plates for 48 h before being challenged with 250 μM APE for various times, as indicated in the experiment. The APE-treated cells were subjected to cAMP assays by following the manufacturer’s protocol for adherent cells in the CatchPoint^®^ cAMP Fluorescent Assay Kit (Molecular Devices, Sunnyvale, CA), modified by changing the forskolin stimulation from 20 μM for 15 min to 10 μM for 10 min.

### Statistical Analysis

The quantitative data are expressed as the means ± S.E. and were analyzed by ANOVA using SPSS 11.0. Post-hoc comparisons of means were made using Scheffe’s or Tukey’s methods, where appropriate.

## Additional Information

**How to cite this article**: He, M. *et al*. GRK5 Deficiency Leads to Selective Basal Forebrain Cholinergic Neuronal Vulnerability. *Sci. Rep.* **6**, 26116; doi: 10.1038/srep26116 (2016).
